# Infection homeostasis: implications for therapeutic and immune programming of metabolism in controlling infection

**DOI:** 10.1007/s00430-015-0402-5

**Published:** 2015-03-24

**Authors:** Konstantinos Kotzamanis, Ana Angulo, Peter Ghazal

**Affiliations:** 1 Division of Pathway and Infection Medicine, Edinburgh Infectious Diseases, University of Edinburgh, Medical School, Edinburgh, Scotland, UK; 2 Institut d’Investigacions Biomèdiques August Pi i Sunyer, University of Barcelona, Barcelona, Spain; 3 SynthSys, University of Edinburgh, The King’s Buildings, Edinburgh, Scotland, UK

**Keywords:** Infection, Glycolysis, CMV, Fatty acid synthesis, Metabolism, Inflammation

## Abstract

Homeostasis underpins at a systems level the regulatory control of immunity and metabolism. While physiologically these systems are often viewed as independent, there is increasing evidence showing a tight coupling between immune and metabolic functions. Critically upon infection, the homeostatic regulation for both immune and metabolic pathways is altered yet these changes are often investigated in isolation. Here, we summarise our current understanding of these processes in the context of a clinically relevant pathogen, cytomegalovirus. We synthesise from the literature an integrative view of a coupled immune–metabolic infection process, centred on sugar and lipid metabolism. We put forward the notion that understanding immune control of key metabolic enzymatic steps in infection will promote the future development of novel therapeutic modalities based on metabolic modifiers that either enhance protection or inhibit infection.

## Introduction

Viruses are extreme obligate parasites that are strictly dependent on the host’s energy and biosynthetic pathways for their replication and morphogenesis, and therefore, these pathways, in particular, afford the host critical immune and non-immune checkpoints to control infection [1]. Notably, the homeostatic regulation of these pathways not only acts to maintain a dynamic equilibrium but also plays a vital role in responding to a wide range of external stimuli, such as infection, which at a systems level must integrate and coordinate multiple diverse processes ranging from the homeostasis of membranes to energy and immunity [2–4]. Using this line of reasoning, we previously put forward the concept for the ‘homeostatic regulation of infection’ which is highly relevant to a wide range of pathogens including cytomegalovirus (CMV) and other members of the herpes virus family that are clinically important and well known to establish lifelong infections [3]. Homeostasis therefore underpins many of the regulatory principles discussed in this review.

Historically, the immune system and metabolism have been intensively investigated at different times and by separated research communities (immunologist and biochemists) leading to an unintended misperception that these act as independent systems. However, there is now increasing evidence to show that these two systems are intimately integrated, share resources and cross-regulate each other [5–10]. While we will highlight some of these recent studies in relation to cytomegalovirus infection, we refer readers to a number of excellent recent reviews that cover the elucidation of the molecular mechanisms linking some of these systems [6–10]. At the cellular level, viral infection adds considerable burden to the cell with the increased demand for energy and metabolic building blocks that are commandeered for the production of new viral progeny. A number of different viruses have been demonstrated to alter the metabolism of infected cells, including, but not limited to hepatitis C virus, rift valley virus, human immunodeficiency virus and human CMV (HCMV) [11–14]. These changes may be pro, or antiviral, or simply bystander effects. In the case of cytomegalovirus infection of macrophages, some of these metabolic alterations, in particular lipid biosynthesis, have been mechanistically linked to the interferon (IFN) response [15, 16].

Here, we provide a discussion of how cytomegalovirus infection modifies metabolism of the host cell, how immune signalling regulates metabolism and how intermediate metabolites can regulate all three processes: infection, the immune response to infection and metabolism itself. Finally, as a perspective for grappling with this new and emerging understanding, we place metabolism at the centre of an immune–metabolic–infection axis that integrates signals from both the host and pathogen. We propose that metabolism provides a highly tunable switch used by both viruses and the immune system to regulate infection. Hence, the lessons learnt from how the immune system regulates metabolism will inform new therapeutic strategies targeting metabolic enzymes to control acute and latent infections.

## Sugar requirement and immune regulation

In normal resting cells, glucose provides the main carbon energy source and is actively imported into cells and rapidly converted to pyruvate in order to enter the tricarboxylic acid cycle (TCA), where it is catabolised to CO_2_ through oxidative phosphorylation and which leads to the production of ATP [17, 18]. These processes of using glucose require oxygen. However, under anaerobic conditions, cells can switch to a more inefficient way of using glucose that converts it to lactate. This leads to a smaller number of ATP production per glucose molecule (two ATP molecules) in comparison with oxidative phosphorylation (36 ATP molecules) [19]. Importantly, this now allows the sugar carbons to be used as building blocks for anabolic processes rather than eliminating through the generation of CO_2_.

**Fig. 1:**
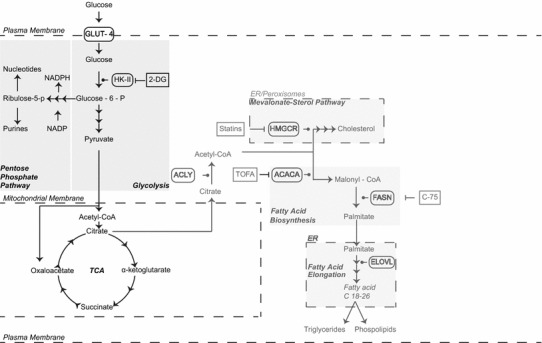
Metabolism of a resting uninfected cell. In a resting cell, glucose is actively imported and used to fuel oxidative phosphorylation. The glycolytic pathway in the cytosol converts glucose to pyruvate. Pyruvate is imported into the mitochondria where it is used to generate NADH and FADH through the TCA cycle. NADH and FADH can be oxidised by the electron transport chain to generate ATP. Citrate generated in the mitochondria can be shuttled out to generate cholesterol and fatty acids when this is required.* Repression arrows* indicate specific drug inhibitors. Glucose-6-P can also be shuttled to the pentose phosphate pathway where it can be used for generation of NADPH, nucleotides and purines. *GLUT* glucose transporter, *TCA cycle* tricarboxylic acid cycle

Indeed, it was Otto Warburg in 1924 that made the key fundamental observation that cancer cells, despite the fact that they have adequate supply of oxygen, utilise anaerobic metabolism [20]. He posited that in order for a cell to divide it needs to increase its biomass by reprogramming the TCA cycle for anabolic purposes [21, 22]. In this scenario, pyruvate (a product of the glycolytic pathway) in the TCA cycle can get converted to citrate that can be shuttled to the cytosol instead of completing the whole TCA cycle (Fig. 1). There it can be converted to acetyl-CoA and be used for the biosynthesis of fatty acids which could be employed to form cell organelles and support division. In fact, a number of studies have shown that as cancer progresses, there is an increased production in fatty acid biosynthesis [23]. A similar process in reprogramming the TCA cycle also occurs during immune stimulation and upon infection of cells.

### Glycolytic and glutaminolysis pathways

#### Virus infection

**Fig. 2:**
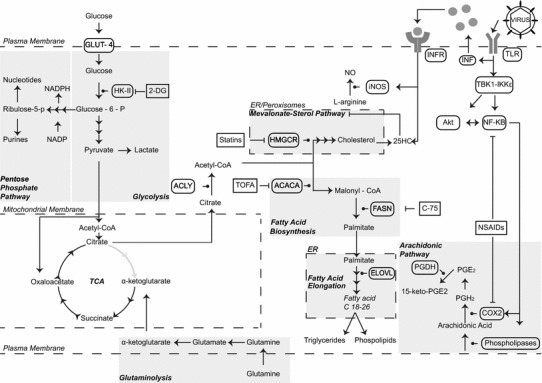
Metabolism of a cell infected by HCMV. A cell infected by HCMV has its metabolism heavily modified. Glucose importation is up-regulated due to up-regulation in the expression of GLUT-4. Glucose imported is converted to pyruvate, which can be oxidised to lactate for the generation of two molecules of ATP. Some of the pyruvate can be imported in the mitochondria and converted to citrate and instead of being used in the TCA is rapidly shuttled out where it is employed for fatty acid biosynthesis. Glutamine importation is required in order to support the TCA cycle through anaplerosis, which is vital to maintain the mitochondrial membrane potential. If the mitochondrial membrane potential is lost, cytochromes get released in the cytosol and caspases are activated leading to apoptosis. The arachidonic pathway is also up-regulated, and there is increased release of arachidonic acid from membranes by lipases, which COX-2 converts to PGH_2_ a precursor for prostanoids. *Repression arrows* indicate specific drug inhibitors. *TLR* Toll-like receptor, *IFN* interferon, *GLUT* glucose transporter, *TCA cycle* tricarboxylic acid cycle, *HK*-*II* hexokinase-II, *Akt* protein kinase B, *NO* nitric oxide, *NSAIDS* non-steroidal anti-inflammatory inhibitory drugs, *PPP* pentose phosphate pathway

A series of systematic metabolomics studies documented that infection of fibroblasts with HCMV dramatically alters the cell metabolome towards fatty acid biosynthesis [14, 24, 25]. A comparison of Figs. 1 and 2 will aid in following the discussion of the changes that occur upon infection. Studies investigating the flux of ^13^C labelling in infected fibroblasts revealed that during infection, uptake of glucose is greatly enhanced and through increased glycolytic activity, carbon atoms are shuttled from glucose to citrate and then to fatty acid biosynthesis [14, 25, 26]. There are multiple regulatory steps involved in this process: according to *McArdle* and co-workers, HCMV activates AMP-activated protein kinase (AMPK), an energy sensor responding to low ATP/ADP ratios in cells, which in turn up-regulates glycolysis [27]; PKR-like endoplasmic reticulum kinase (PERK), an ER-associated stress sensor protein [28] is induced; the sterol regulatory protein 1 (SREBP-1), a transcription factor for regulating fatty acid biosynthesis is up-regulated at late times of infection [29]; and expression of Glut-4, a glucose transporter (GLUT), and carbohydrate-response element-binding protein (ChREBP), a transcriptional factor that regulates glucose metabolism and lipogenesis [30, 31] are induced during HCMV infection. All these alterations lead collectively to higher glucose uptake and increased lipogenesis [29–31]. Along with changes in glycolysis, the virus also increases glutaminolysis, and glutamine gets converted to α-ketoglutarate and through anaplerosis allows the TCA cycle to function [32]. Thus, while in normal diploid fibroblasts glutaminolysis is not required, it is essential for infected cells, and blockade of glutaminolysis significantly impacts on the viability of HCMV-infected cells [32]. Finally, Seo and Creswell [33] suggest that this cascade may also be regulated by viperin. Viperin is an IFN-stimulated multifunctional antiviral protein that gets hijacked by viral mitochondrial inhibitor of apoptosis (vMIA), a HCMV protein, resulting in alteration of its function [33–35]. HCMV appears to up-regulate these pathways and inhibition of them and/or of fatty acids synthesis leads to reduction in viral progeny [11, 25, 36]. In fact, the concept of inhibiting metabolic pathways to block CMV infection it is not so new. As early as 1981, 2-deoxy-d-glucose (2-DG), a glycolysis inhibitor, was shown to potently inhibit viral replication [37]. A schematic of these alterations is shown in Fig. 2.

#### Immune activation

**Fig. 3:**
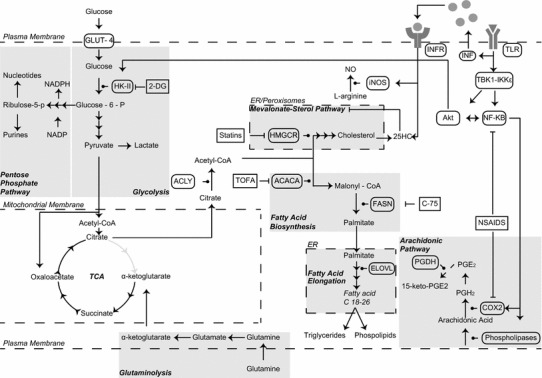
Metabolism of a macrophage activated by immune signals. Upon activation of TLRs, the cell releases downstream IFN and at the same time, TBK1-IKKε kinases are activated leading to iNOS, Akt and NF-KB activation. This leads to increased glycolysis through activation of HK-II. NO production is also up-regulated from iNOS and blocks generation of ATP by mitochondria, and this also leads in up-regulation of glycolysis in order to produce ATP from pyruvate fermentation. Part of the pyruvate produced by glycolysis is imported to mitochondria where it gets converted to citrate and shuttled to the cytosol and used for fatty acids synthesis required by the cell for its activation. At the same time due to direct signalling by IFN, the mevalonate–sterol pathway is down-regulated. In the arachidonic pathway, COX2 is activated by NF-KB and prostanoids produced by are used as signals in an autocrine and paracrine way to regulate the immune response.* Repression arrows *indicate specific drug inhibitors. The key differences to note in comparison Fig. 2 are TLR activation alone involving HK-II activation by Akt and that TLR agonist is the activator and not virus. *TLR* Toll-like receptor, *IFN* interferon, *GLUT* glucose transporter, *TCA cycle* tricarboxylic acid cycle, *HK*-*II* hexokinase-II, *Akt* protein kinase B, *NO* nitric oxide, *NSAIDS* non-steroidal anti-inflammatory inhibitory drugs, *PPP* pentose phosphate pathway

The question arises as to whether these changes upon infection are in isolation of immune activation. A comparison of Fig. 3 with 1 will aid in following the discussion of the changes that occur upon immune activation. In this regard, it is now emerging that immune cell activation also leads to marked detectable metabolic changes in cells [38]. Upon activation, lymphocytes, macrophages and dendritic cells have all been shown to alter dynamically their metabolism [39–42]. The precise metabolic changes that occur differ not only from cell type to cell type, but also between different phenotypes of the same cell type [43]. For the purpose of this review, we will focus on discussing macrophages and dendritic cells (DCs) that are permissive to CMV and play a key role in viral dissemination and latency. A schematic summary of these changes is shown in Fig. 3.

Immune-activated dendritic cells and macrophages show enhanced glycolysis and are further compromised in their ability to sustain oxidative phosphorylation in the presence of immune-activated nitric oxide (NO) production, as this molecule potently inhibits oxidative phosphorylation in the mitochondria [44]. Furthermore, in the case of immune-activated macrophages, the reduced mitochondrial respiration is also highly effective at augmenting the generation of anti-infective reactive oxygen species (ROS) [45]. In the case of resting DCs, oxidative phosphorylation is mainly used for energy purposes. However, when treated with Toll-like receptor agonists, these cells adopt a Warburg style metabolism and after 12 h of activation, mitochondrial oxygen consumption ceases and glucose uptake is increased even though it is not using the TCA cycle for ATP production [44, 46]. As described above, this change allows DCs to survive the production of NO and when DCs are treated with 2DG at early stages of activation (before 6 h), the development of the activated phenotype is inhibited [44, 46]. More recent studies reveal that this Toll-like receptors (TLR) activation supports anabolic demands of DCs which include biosynthesis of fatty acids through the shuttling of carbon atoms from glucose to TCA cycle and then de novo synthesis of fatty acids and prostaglandins [47]. Similarly, IFN-activated macrophages are highly glycolytic and also adopt a Warburg style metabolism [42]. Much like DCs, macrophages shuttle glucose carbon to fatty acids and lipids synthesis, and at the same time, their pentose phosphate pathway (PPP) flux is increased [42]. These changes are required for meeting the demands for enhanced production of membranes in the ER and golgi to accommodate the stimulated protein production as part of the immune-activated functions, including proteins for antigen presentation and cytokine production.

A critical part of this metabolic reprogramming is the very rapid (within minutes) accumulation and secretion of succinate, a metabolic intermediate of the TCA cycle, that has been shown to act as a signalling molecule for the activation of the alpha subunit of hypoxia-inducible factor-1 (HIF-1a) and the induction of HIF-1a-dependent genes [48, 49]. A further interesting aspect of the crosstalk that occurs between metabolism and immunity is the availability of substrates that may also alter the phenotype of macrophages. For instance, overexpression of GLUT-1 leads to increases in glycolytic flux that drives a pro-inflammatory M1-like phenotype by macrophages without any other signalling [50].

Altogether, broadly comparing the isolated infection and immune activation studies, it appears that the alterations in energy and central carbon metabolism show metabolic cooperation, despite different cell type-based investigations. The apparent parallel trajectories of infected and immune-activated cells (compare Figs. 2, 3) strongly suggest that rather than the virus driving these changes, it has the option for co-opting at early times of infection the TLR-mediated signalling of metabolism. It will be important in the future to determine whether CMV-infected macrophages also exhibit a similar alteration in flux and metabolic functions.

## Lipogenic requirement and immune regulation

Lipids are a diverse family of chemically distinct molecules that can be hydrophobic or amphipathic and serve multiple roles in a cell. They are used to store energy, signal and maintain cellular structure and have a key building role for all membranes and organelles [51] including for the growth of viruses. For instance, an important class of non-steroidal lipids that act as signalling molecules in immunity are retinoids, which are also known to directly and indirectly control HCMV and MCMV gene expression and viral replication [52–54].

**Fig. 4:**
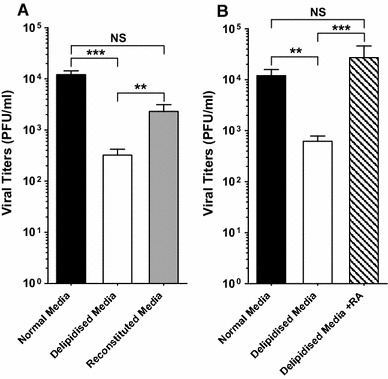
Lipogenic factor requirement for MCMV growth. **a** NIH 3T3 cells are pre-treated with normal (DMEM 3 % CS), delipidised (DMEM 3 % charcoal resin-treated CS) or reconstituted (DMEM 3 % charcoal resin-treated CS containing chloroform–methanol serum extracted lipids) medium for 4 h and then infected with MCMV at a multiplicity of infection (MOI) of 0.02. After infection, cells received the corresponding medium (normal, delipidised or reconstituted). Viral titers are determined by plaque assay at day 7 after infection from culture supernatants. *n* = 4, data are mean ± SD. **b** NIH 3T3 cells are pre-treated with normal, delipidised or 10 μM 9-*cis* RA in delipidised medium for 4 h and then infected with MCMV at an MOI of 0.01. After infection, cells received the corresponding medium (normal, delipidised without or with 10^−9^ M 9-*cis* RA. Viral titres are determined as in **a**. *n* = 3, data are mean ± SD. **p ≤ 0.01; ***p ≤ 0.001, determined with an unpaired Student’s *t* test

Figure 4 shows the results of very early experiments that we conducted to directly test a potential lipogenic requirement for CMV growth in cells. For these studies, fibroblasts were incubated in medium supplemented with charcoal-treated serum (delipidised media), in which biologically active lipid factors had been removed, and were subsequently infected with murine CMV (MCMV) and analysed for viral production. As shown in Fig. 4, the growth of CMV was statistically strongly attenuated under these lipid-depleted conditions. It is worth noting that cell viability was not significantly affected by the removal of lipids in the culture medium in the conditions of the assay. We next asked whether a pro-viral lipogenic component in the serum was possibly removed. To test this notion, a chloroform–methanol method for removing lipids was used [55] whereby neutral lipids (triglycerides, waxes and pigments) are extracted by chloroform and phospholipids; glycoproteins and cholesterol are removed by methanol. These extracted lipids were subsequently used to reconstitute the delipidised serum. Notably, we found that addition of the reconstituted lipids to the delipidised media substantially reversed the viral growth defect exhibited after lipid depletion (Fig. 4a). It is possible that the delipidised media conditions simply impacts the cell by reducing an essential ‘lipid’ resource for the production of viral membranes, or alternatively but not mutually exclusive, is the possibility for a pro-viral regulatory lipogenic factor. In order to distinguish between these possibilities, we sought to test whether the capacity of infected cells to generate infectious particles under delipidised media conditions is due to limited membrane lipid resources. In order to assess the maximal capacity for additional virus production under delipidised culture conditions, we employed a well-established and potent regulatory lipid for enhancing CMV, 9-*cis* retinoic acid (RA) [54]. Treatment of cultures in delipidised medium with 9-*cis* RA led to a notable increase in MCMV growth that reached levels comparable or even higher than those obtained in cultures containing the normal medium, indicating that resources are not limiting the capacity of the cell to produce virus (Fig. 4b). Thus, these investigations reveal a requirement of lipogenic factors for optimal MCMV infection and indicate that this dependency is not only due to a deficit of membrane resources for viral growth in the cells. Please note that we do not infer from these experiments that retinoic acid is the regulatory limiting lipogenic component although this possibility cannot be excluded. Most importantly, these experiments underscore that careful consideration should be given to interpreting experiments that block or deprive cells of lipids and assessing the impact on viral growth. For this reason, the view that a pro-viral host lipid represents a depleted resource may not necessarily be the case and should be distinguished from possible effector or regulatory signalling functions. In the next section, we discuss the functional roles and alterations that occur for lipids upon infection and immune activation.

### Long chain fatty acid pathway

#### Virus infection

As described above, HCMV infection shuttles carbon atoms to fatty acid synthesis (compare Figs. 1, 2). Fatty acids are carboxylic acids with long aliphatic tails. Fatty acid synthesis begins in the cytosol with the conversion of acetyl-CoA to malonyl-CoA by acetyl-CoA carboxylase (ACACA), the committing step in fatty acid biosynthesis (Fig. 1). Fatty acid synthase (FASN) then uses malonyl-CoA to add two more carbon atoms to the aliphatic chain of a fatty acid. Fatty acids are elongated up to 16 carbons from FAS, and then, they can be elongated further in the ER by the elongation system in which fatty acid elongases (ELOVLs) play a vital role [56] (Fig. 1).

Drug inhibition of ACC with TOFA and of FASN with C-75 or other inhibitors have shown marked inhibition of HCMV replication [25, 36]. HCMV has also been shown to up-regulate ACC expression along with a number of other enzymes important for fatty acid biosynthesis. More recent studies have demonstrated that the virus requires very long chain fatty acids (VLCFAs) to replicate effectively [11]. Inhibition of specific ELOVLs attenuated the replication of the virus, and the fact that the envelope of the virus in untreated fibroblasts was strikingly enriched for those specific fatty acids, suggesting that indeed the requirement for this pathway may act at the viral membrane level. In further support of this, it was also noted that inhibition of the pro-viral ELOVLs does not significantly reduce the number of virions, but instead greatly reduces infectivity. This was attributed to the ability of those VLCFAs to produce an envelope with correct curvature or other physicochemical properties necessary to bud virions from cells [11]. In this connection, phospholipids are essential membrane structural lipids consisting of a hydrophobic fatty acid tail. For HCMV, the glycerophospholipid composition of the virus membrane has been found to be very similar to synaptic vesicles suggesting a re-purposing or modelling of the host lipid membranes for its morphogenesis [57]. HCMV in general dramatically remodels the structure of a cell, leading to the gross enlargement of infected cells (cytomegalic) and the formation of a new compartment termed the virion assembly compartment (VAC) [58]. VAC is a kidney-shaped structure adjacent to the nucleus that is crucial for HCMV replication and is formed by extensive remodelling of the secretory membrane apparatus [59–61].

#### Immune activation

The immune activation of cells also impacts on lipid and fatty acid metabolism [62–64], see Fig. 3 for a schematic summary of pathways. As described above, macrophages and DCs of specific phenotypes promote de novo fatty acid synthesis upon activation [47, 65]. Although still not well understood, these changes appear to be important for the activated function of the cell; for instance, inhibiting fatty acid synthesis can impair phagocytosis [65]. An activated macrophage leads to significant changes in phospholipids and fatty acids that promote the formation of filopodia and the biogenesis of new organelles such as mitochondria, lysosomal compartments, and expanded ER and golgi [47, 65, 66]. These metabolic changes are therefore essential for activated macrophages and dendritic cells to be transformed, from small quiescent cells to cells that are actively phagocytic, present antigens and secrete a huge number of cytokine and immune regulatory molecules.

Altogether, these immune-driven changes appear to have similarity to those observed in infected cells despite the studies being conducted in different cell types and point to a more unified response (compare Figs. 2, 3). This raises again the question of whether immune activation initiates the metabolic changes that upon infection also opportunistically favour viral growth. In this scenario, such immune-initiated re-programming of metabolism may be required and co-opted by the virus but alone may not be sufficient. Future studies should address whether it is likely that additional metabolic state changes are further needed to encourage viral growth, and we speculate that viral gene products may have a contributory role in governing metabolic programming in the infected cell.

### Prostanoid biosynthesis pathway

#### Virus infection

As mentioned at the start of the lipid section, not all lipids will necessarily have a structural role as membrane lipids but may also contribute regulatory functions or signals that promote or inhibit viral growth. In this regard, an interesting class of signalling lipids that HCMV appears to depend on are eicosanoids. These are produced by oxidation of 20 carbon fatty acids and play critical regulatory signalling roles in immunity and inflammation. Upon infection of fibroblasts, HCMV up-regulates the expression of genes related to the eicosanoids pathway [67, 68]. Among them are cytosolic phospholipase A2 (cPLA2) and cyclooxygenase-2 (COX-2) that are responsible for the generation of prostaglandin H2 (PGH2) acid from cell membranes and its conversion to prostaglandin H2 (PGH_2_) [69–71]. The inhibition of COX-2 has been shown to reduce HCMV progeny while the introduction of prostaglandin E2 (PGE_2_) rescues the drug blockade [71]. While the antiviral mechanism for inhibition is not clear, PGE_2_ has been reported to stimulate the activity of the virus major immediate early promoter [72]. A later report from Schröer and Shenk [71] demonstrated that inhibition of COX-2 also blocks cell-to-cell viral spread although the precise mechanism has not been defined.

In addition to the studies indicating that blocking prostaglandin synthesis inhibits viral growth, we have previously reported through evaluation of a highly selective and chemically diverse synthetic prostanoid library that the replication of MCMV can be enhanced or strongly curtailed depending on the specific prostanoid chemical structure [73]. Thus, altogether these studies suggest key regulatory functions for prostanoid signalling in determining the outcome of a viral infection. In this context, we have been successful, as a proof-of-principle study, to identify a potential therapeutic prostanoid that had potent anti-CMV activity [73].

#### Immune activation

The inflammatory induction of prostaglandins and other related lipids by macrophages and DCs have a critical secondary autocrine and paracrine signalling role in immunity [70, 74, 75]. Notably, lipidomic studies of the macrophages have revealed that upon activation with a selective TLR4 agonist, a rapid increase in eicosanoid synthesis takes place [76]. Depending on factors such as the nature of the signalling or length of exposure to it, different outcomes on lipid alterations have been reported in macrophages and DCs [77, 78]. Moreover, differential gene regulation of enzymes that participate in the eicosanoid biosynthesis pathway has been documented in pro-inflammatory (M1) and anti-inflammatory (M2) polarised macrophages [79]. Thus, IFN and LPS activation of M1 macrophages potently induced COX-2, and down-regulated COX-1 and other pathway-related enzymes, such as leukotriene A4 hydrolase. In contrast, M2 macrophages activated with IL-4 exhibited an up-regulation of COX-1. TLR treatment of macrophages induced the microsomal isoform of PGE synthase (mPGES). On the other hand, Th2-associated cytokines IL-4 and IL-13 down-regulated the expression of mPGEs in macrophages [80].

In turn, prostanoids released from macrophages and DCs participate in autocrine loops to regulate COX-2 expression. In macrophage cells, PGE2 has been reported to induce COX-2 and mPGES-1 [81]. In addition, released prostanoids are able to affect, either in a positive or in a negative manner, a number of macrophage and DC functions [78]. As an example, PGE2 has been shown to usually exert inhibitory actions on macrophages and DCs, such as suppressing MHC class II expression and TNF-α production and inducing IL-10 expression [82–84]. However, PGE2 has also been documented to exert stimulatory effects on these two cell types, including the induction of IL-23 in DCs through cooperation of PGE2 with CD40-mediated signalling [85]. Most significantly, PGE2 release from myeloid cells has been shown to restrain T cell activation, and clinical trials have revealed that blockade of PGE2 synthesis exacerbates severe systemic inflammation. In this connection, it has also been suggested that PGE2 released from HCMV-infected macrophages may contribute to the immunosuppressive activity of HCMV [86]. Future studies elucidating the regulatory role of PGE2 in immunity will significantly advance our understanding of lipid host protection pathways modulated in cytomegalovirus disease.

### Mevalonate–sterol pathway for cholesterol biosynthesis

#### Virus infection

Cholesterol is known to have a causal link to inflammatory diseases and its oxidised metabolites (oxysterols) alter adaptive and innate immunity, and innate immune signalling has been shown to modulate the dynamics of cholesterol homeostasis [62–64, 87]. In this connection, over the last century, natural and experimental infection studies have reported for a wide range of pathogens a state of hypocholesterolemia during the acute phase (2–4 days) of infection that is often followed by a rebound period of hypercholesterolemia [88–96]. Currently in the critical care of sepsis, monitoring of cholesterol levels is used as a biomarker for prognosis. Although blood cholesterol levels for CMV infection have not been measured, acute hepatic infection by MCMV has been shown to increase total cholesterol levels in the liver [97]. Hence, the biosynthesis and metabolism of cholesterol takes centre stage in infection biology. Work from Alwine et al. [29] suggest that HCMV induces adipocyte-like lipogenesis and overrides normal sterol feedback controls in order to maintain high levels of constitutive lipid synthesis during infection.

Cholesterol biosynthesis starts with acetyl-CoA whereby the reductase, HMGCR, represents a critical regulated step in the mevalonate arm of the sterol biosynthesis pathway (Fig. 1). The pathway is responsible not only for the production of cholesterol but also for generating immune and sex steroid hormones while the upstream mevalonate arm of the pathway produces metabolites used in a diverse array of biological processes including the synthesis of ubiquinone for mitochondrial aerobic respiration with functions in the electron transport chain, and other isoprenoids required for the N-gylcosylation and lipidation of proteins (farnesylation and geranylation of proteins). Thus, the inhibition of HMGCR by statins not only reduces cholesterol but also has the ability to affect multiple products of the mevalonate side branch. Perhaps not surprisingly statins are potent inhibitors for human and murine CMV [15, 98–100], and we were the first to show that the antiviral activity of statin on CMV can be metabolically reversed by mevalonate but not cholesterol treatment [15]. Recent studies have since independently confirmed the inhibition of HCMV by statins and metabolic rescue of antiviral action by using mevalonate [98]. The metabolic rescue experiments unequivocally demonstrate the absolute requirement for mevalonate–sterol biosynthesis pathway for optimal CMV growth. In a series of systematic loss of function and drug inhibition assays, we have provided further evidence implicating mevalonate-derived products such as isoprenoids involved in the lipidation of proteins for CMV growth [16, 101]. Inhibition of the mevalonate–sterol arm does not affect the binding or entry of CMV but evidence to date points to the suppression of the major immediate early gene expression as well as blocking cell-to-cell spread [16].

It is important to note that while these studies indicate that cholesterol alone is not the primary metabolite for statin antiviral action, they do not rule out an important biological requirement for cholesterol in the CMV life cycle. In this regard, studies evaluating the proteomes of fibroblasts cells before and after HCMV infection by mass spectroscopy identified LRP1, an LDL receptor-related protein involved in cholesterol uptake, as a transiently increased protein at 24 h after infection. In human fibroblasts, knockdown of LRP1 was found to enhance HCMV infectivity and also lead to increased cholesterol in released virions. Importantly, the cholesterol content of the virus envelope was shown to be critical for HCMV fusion with the host cell. These results indicate that increases in cellular and virion cholesterol content lead to more efficient fusion of the virion envelope with the plasma membrane and therefore increased virion infectivity [102].

Upon infection of macrophages with MCMV, we found by metabolic and transcriptional profiling studies that one of the most statistically significant pathways suppressed upon infection was mevalonate–sterol metabolism. The reasons for this reduction are complex and may not be identical or equivalent in all cell types and media conditions as this pathway is directly regulated by the immune response and by extracellular cholesterol. For instance in macrophages but not fibroblasts, we find a highly specific and dramatic increase in the production of an oxidised cholesterol metabolite generated by the hydroxylase CH25H. The regulation of CH25H appears to be highly cell type and tissue specific and is mainly restricted to immune (myeloid and lymphoid) and to certain epithelial cell types. We, and others, identified and showed that the metabolic product of CH25H enzyme, 25-hydroxycholesterol (25HC), a well-characterised inhibitor of the mevalonate–sterol biosynthesis pathway, acts as a potent antiviral regulatory factor by principally suppressing the isoprenoid branch of the pathway and through affecting cellular membranes [16, 66, 103]. It remains an open question if CMV encodes viral proteins that counter these inhibitory effects. Previous studies from Alwine et al. [29] have suggested that HCMV infection of fibroblast can override normal sterol feedback control in order to maintain high levels of constitutive lipid synthesis during infection.

Overall, these studies underscore that the interplay between immune activation, infection and metabolic changes cannot be considered in isolation.

#### Immune activation

Similar to the early infection studies examining blood cholesterol, in completely unrelated early investigational studies of recombinant IFN treatment in humans, for a range of different disease indications, a transient drop in blood cholesterol levels has also been repeatedly documented. Radiolabelled metabolic studies have further indicated that this particular side effect of IFN treatment is due to a suppression of endogenous sterol synthesis [94]. Strikingly, mechanistic and functional physiological linkages between the observational based infection and IFN studies have not, until recently, been investigated or considered. IFN and TLR activation are now known to coordinate the reduction in cholesterol biosynthesis for human and murine macrophages [10, 15, 66, 76, 104]. We demonstrated that IFN-induced inhibition of sterol biosynthesis serves as an integral component of the very early cellular response to virus infection [15]. Metabolic profiling [15] and molecular screening [103] studies led ourselves and others to identify IFN-elicited 25HC as an important effector of the IFN-induced antiviral response in macrophages [15, 16]. This lipid has emerged to have broad-spectrum antiviral and immune modulatory functions [10].

We demonstrated that CMV infection leads to a TLR-induced type I IFN-mediated down-regulation of the master transcription factor for sterol biosynthesis, SREBP2 [15]. More recently, we have also shown that IFN signalling specifically targets in a SREBP-independent manner the statin-targeted reductase, HMGCR, for rapid proteosomal degradation that is strictly coupled to the endogenous synthesis of 25HC [105]. Our studies of immune-activated macrophages identified STAT1 transcriptional regulation of the cholesterol hydroxylase, CH25H, as a direct mechanistic link between IFN-mediated antiviral activity and the regulation of sterol metabolism [16]. Investigations of SREBP2-dependent IFN effects have implicated the sterol biosynthesis pathway involved in both antiviral and anti-inflammatory pathways [16, 106]. Further studies are required to more fully understand the role of SREBP2 and the mevalonate–sterol pathway in immunity and infection.

## Therapeutic perspective

Here we have discussed how cytomegaloviruses are critically dependent on and co-opt many aspects of cellular sugar and lipid metabolism to facilitate their life cycles across species and how the inhibition of these processes can curtail virus replication. Overall, we find a remarkable level of metabolic cooperation between infection and immunity. Defining how immunity and metabolism are integrated, share resources and cross-regulate one another during infection is therefore an essential yet incompletely understood area of infection biology. Notably, unbiased systems biology approaches are providing valuable new insights into this integrative complexity and computational studies based on predictive modelling will be essential in the future functional design and control of an immune–metabolic–infection axis [107].

**Fig. 5:**
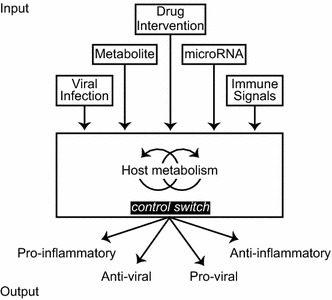
Metabolism acts as a tunable switch device that integrates multiple input signals to direct a range of biological outputs

In this regard and to advance this future perspective, Fig. 5 summarises and shows as a schematic representation of the inputs and outputs for this axis, with metabolism positioned in the middle connecting in an integrative manner the various distinct biological processes. Conceptually, this diagram extends our previous ‘cellular clockwork model of infection’ [1] that underscored the dependency of pathogens on cellular pathways as a means for therapeutic drugs or the immune system to control infections. Here we extend the previous model to emphasise, at a systems control level, an important design principle suggesting that cellular metabolism behaves as a ‘tunable device’ and that the control of metabolic flux in these pathways is necessary for driving the range and type of biological response (Fig. 5).

While the concept of metabolism acting as a ‘tunable device’ for governing cellular functions is new, it remains speculative yet testable for the future. Thus, we believe that further understanding and identifying the host molecular circuitry for new and recently discovered immune-regulated metabolites and enzyme pathway systems will aid in the development of new therapeutic innovations to prevent infection (as an anti-infective) or enhance immunity (as adjuvant-based metabolic modifiers).

Although we have focussed our discussions on sugar and lipid pathways, these are not the only examples of an immune–metabolic–infection axis known to control infections. In particular, the immune-activated metabolism of amino acids has been long established. Stimulation of macrophages with IFN results in the rapid induction of the tryptophan-catabolising enzyme indoleamine-2,3-dioxygenase resulting in broad-spectrum antimicrobial and immunosuppressive effector functions. Other examples include the immune-regulated activities of the arginine-hydrolysing enzymes nitric oxide synthase 2 (iNOS) and arginase 1 (Arg1). In the case of these enzyme systems, NO production by iNOS acts as an important pro-inflammatory and antiviral mediator, while Arg1-expressing macrophages contribute to the resolution of inflammation and wound repair. In the context of viral infections, expression of these enzymes can result in a variety of outcomes for the host [108]. Interestingly, HCMV infection of fibroblast is capable of effectively impairing this response [109] and might suggest a similar mode of action for blocking the IFN-induced suppression of sterol biosynthesis.

Strikingly, the glycolytic and fatty acid metabolism changes that favour CMV growth in fibroblasts, parallels the metabolic profile of the pro-inflammatory immune-activated macrophages, but not the anti-inflammatory macrophages. In future, it will be important to determine whether the metabolic cooperation in glycolytic and fatty acid pathways also occurs upon CMV infection of macrophages. Since CMV remains latent in monocytes and reactivates upon immune activation and differentiation, it is plausible that CMV may also exploit these different metabolic stages of the macrophage phenotypes to remain latent and reactivate when conditions favour replication. If so, then therapeutic targeting (‘tuning’) of metabolism in latently infected monocytes may provide a means for curing or eliminating latently infected cells.
